# Quantitative Analysis of Spatial Distribution of Medial Fracture Lines in Intertrochanteric Fractures

**DOI:** 10.1155/abb/6959877

**Published:** 2025-04-10

**Authors:** Miaotian Tang, Hao Zhou, Gaoxiang Xu, Dake Tong, Cheng Xu, Jiantao Li

**Affiliations:** ^1^Department of Trauma and Orthopedics, Peking University People's Hospital, No. 11 Xizhimen South Street, Xicheng District, Beijing 100044, China; ^2^Department of Orthopedics, The Fourth Medical Center of Chinese PLA General Hospital, No. 51 Fucheng Road, Beijing 100048, China; ^3^National Clinical Research Center for Orthopedics, Sports Medicine and Rehabilitation, No. 51 Fucheng Road, Beijing 100048, China; ^4^Department of Orthopedics, First Affiliated Hospital of Zhengzhou University, Zhengzhou 450042, Henan, China; ^5^Shanghai Key Laboratory of Orthopaedic Implants, Department of Orthopaedic Surgery, Shanghai Ninth People's Hospital, Shanghai Jiao Tong University School of Medicine, Shanghai 200011, China

**Keywords:** digital orthopaedics, intertrochanteric fracture, intertrochanteric fracture classification, medial fracture line

## Abstract

The integrity of the medial wall of the proximal femur is crucial for maintaining mechanical homeostasis. However, the impact of intertrochanteric fractures on the medial wall and the optimal diagnostic methodology remain unclear. We retrospectively analyzed CT data from 205 patients with intertrochanteric fractures. The lowest point of the medial fracture line was marked, and a standard coordinate system was established to record its spatial position. The association between AO, Evans, or Tang classification and different types of medial wall disruption was analyzed using Spearman correlation. The lowest point of the fracture line was located in the first quadrant of the proximal medial wall in 20 patients, with spatial coordinates of (6.44 ± 5.47, 6.14 ± 2.71). In 21 patients, it was in the second quadrant, with coordinates of (−7.23 ± 5.86, 8.31 ± 6.59). In 122 patients, it was in the third quadrant, with coordinates of (−9.59 ± 4.32, −24.43 ± 15.79), and in 42 patients, it was in the fourth quadrant, with coordinates of (8.18 ± 4.56, −18.20 ± 12.92). The Tang classification showed a stronger correlation with fracture instability (*r* = 0.40, *p*  < 0.001) compared to the AO (*r* = 0.32, *p*  < 0.001) and Evans (*r* = 0.38, *p*  < 0.001) classifications. The medial wall of the proximal femur is significantly compromised in intertrochanteric fractures, with varying mechanical stability depending on the fracture type. The Tang classification effectively differentiates these stability differences, providing valuable guidance for clinical practice.

## 1. Introduction

With the rapid growth of the aging population and increased life expectancy, intertrochanteric fractures associated with osteoporosis have become increasingly prevalent. Although current surgical technologies and implant systems have demonstrated promising outcomes in the treatment of intertrochanteric fractures, ~20% of patients still experience treatment failure [1]. Consequently, the clinical management of intertrochanteric fractures continues to present significant challenges for orthopedic surgeons.

The reconstruction of the anteromedial cortical support is essential for restoring the integrity and mechanical stability of intertrochanteric fractures [2, 3]. The extent of collapse in the medial cortical region significantly influences the degree of fracture stability, with varying patterns of medial support loss resulting in distinct biomechanical consequences [4–6]. A moderate certainty ratings demonstrated that medial cortical support reduction quality was associated with failed internal fixation [7]. Retrospective clinical analyses demonstrated a significant complication rate of 27% in cases where medial cortical reconstruction was not performed [8].

Preoperative accurate assessment of medial wall damage is crucial for determining optimal surgical strategies in intertrochanteric fracture management. Biomechanical evidence has consistently demonstrated the superior importance of the medial wall in maintaining structural stability compared to the lateral wall. Nie et al. [9] reported significantly different failure loads between lateral and medial wall defects, with mean values of 1596.78 ± 273.17 N and 476.05 ± 138.85 N, respectively (*p*  < 0.001). Yang et al. [10] demonstrated that lateral wall defects exert a biomechanical impact on fracture stability equivalent to 36.6% of the destabilizing effect induced by medial wall defects. Furthermore, comprehensive 3D fracture mapping analyses revealed substantial morphological variations in medial wall fracture patterns [11]. These findings collectively underscore the critical role of medial wall assessment and reconstruction in achieving optimal clinical outcomes for intertrochanteric fracture treatment.

Despite extensive research in this field, previous investigations have failed to quantitatively analyze the spatial orientation and trajectory patterns of medial wall fracture lines or systematically evaluate the diagnostic accuracy of existing fracture classification systems. To address these critical knowledge gaps, we conducted a comprehensive radiographic analysis of 205 consecutive intertrochanteric fracture cases. The primary objectives of this study were twofold: (1) to precisely quantify and characterize the spatial distribution patterns by mapping the lowest point of the medial wall fracture line; (2) to perform a comparative evaluation of the diagnostic efficacy among three widely used classification systems (AO, Evans, and Tang classifications) based on their ability to accurately characterize medial wall integrity.

## 2. Materials and Methods

### 2.1. Patient Inclusion and Exclusion Criteria

Inclusion criteria: (1) low-energy trauma; (2) preoperative hip CT scan; (3) age ≥ 45 years. Exclusion criteria: (1) femoral head necrosis; (2) severe osteoarthritis or rheumatoid arthritis of the hip; (3) hip deformity; and (4) history of contralateral hip fracture. Based on these criteria, 205 patients were included in this study, which was approved by the Institutional Review Board (S2020-114-01).

### 2.2. Reconstruction of the Proximal Femur

Original DICOM files were collected for each patient, and Mimics software was used to reconstruct all three-dimensional fracture fragments. The “Move” and “Rotate” functions in 3-Matic were used to restore each fracture fragment to its natural position in the three-dimensional view, allowing clear observation of the medial wall damage.

### 2.3. Quadrant Division of the Proximal Femoral Medial Wall

**Figure 1:**
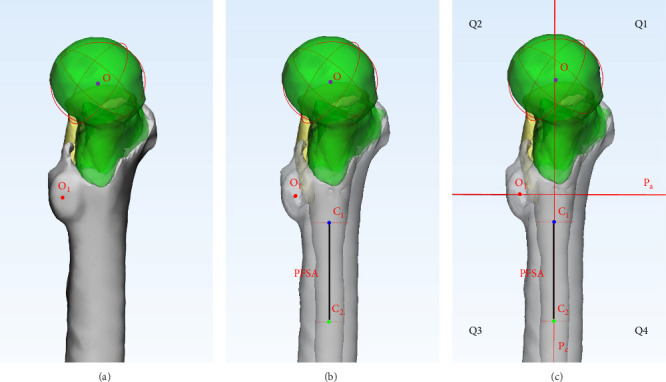
Quadrant division of the medial wall of the proximal femur. (A) Spherical fitting of the femoral head and lesser trochanter; (B) fitting of the proximal femoral shaft; and (C) quadrant division of the medial wall of the proximal femur.

The spherical fitting function in 3-Matic was used to fit the femoral head and lesser trochanter. The marked regions of the femoral head and lesser trochanter surfaces were encompassed >80% to obtain the centers of the fitted spheres (O and O_1_). The tolerance parameter of sphere was set as 0.01 mm in 3-Matic. The lower edge of the lesser trochanter was identified, and the femoral shaft 5 cm below this edge was fitted as a cylinder to obtain the proximal femoral shaft center C_1_ and C_2_. The tolerance parameter of cylinder was set as 0.01 mm in 3-Matic. The plane P_c_ formed by points O, C_1_, and C_2_ was defined as the coronal plane of the proximal femur. The plane P_a_, perpendicular to P_c_ through O_1_, was defined as the transverse plane of the proximal femur. P_c_ and P_a_ divided the medial wall into four quadrants: anterior–superior, posterior–superior, posterior–inferior, and anterior–inferior, labeled as Quadrant 1 (Q1), Quadrant 2 (Q2), Quadrant 3 (Q3), and Quadrant 4 (Q4), respectively (Figure 1). In Q1, both the abscissa *X* and the ordinate *Y* are positive. In Q2, the abscissa *X* is negative, while the ordinate *Y* is positive. In Q3, both the abscissa *X* and the ordinate *Y* are negative. In Q4, the abscissa *X* is positive, while the ordinate *Y* is negative.

Spherical fitting (femoral head and lesser trochanter).

Surface selection:

Cylindrical fitting (femoral shaft).

Surface Selection: Marked 5 cm segment distal to the lesser trochanter, excluding osteophytes or irregular cortical surfaces.

### 2.4. Quantification of the Spatial Position of the Fracture Line's Lowest Point

**Figure 2:**
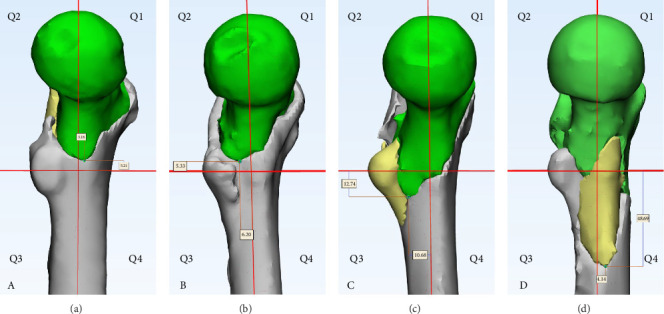
Definitions of different quadrant distributions in intertrochanteric fractures. (A) The lowest point of the fracture line is located in Q1; (B) the lowest point of the fracture line is located in Q2; (C) the lowest point of the fracture line is located in Q3; and (D) the lowest point of the fracture line is located in Q4.

We define the lowest point of the fracture line as point D. The distance from point D to the coronal plane P_c_ is defined as the value of the abscissa *X*. The distance from point D to the transverse plane P_a_ is defined as the value of the ordinate *Y*. The positive or negative values of the coordinates are determined based on the quadrant in which point D is located. Through this method, we describe the spatial distribution of the lowest point of the fracture line within the coordinate system (Figure 2).

### 2.5. Diagnostic Performance

Mechanical stability of fractures decreases from Q1 to Q4. Based on this, we evaluated the diagnostic efficacy of AO [12], Evans [13], and Tang classifications [14] in assessing the mechanical stability of intertrochanteric fractures, specifically their consistency with postoperative proximal femoral stability.

### 2.6. Statistical Analysis

IBM SPSS 21.0 was used for statistical analysis. The Shapiro–Wilk test was used to determine if the data were normally distributed, with normally distributed data presented as mean±standard deviation. Spearman's rank correlation was used to analyze the correlation between AO, Evans, and Tang classifications and the stability of different medial wall fracture types. A *p*-value < 0.05 was considered statistically significant.

## 3. Results

A total of 205 patients with intertrochanteric fractures were included, with an average age of 65 years.

### 3.1. Quantitative Characteristics of the Medial Fracture Line

**Figure 3:**
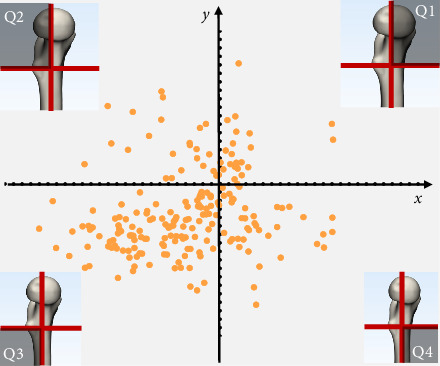
Distribution of fracture line quadrants in 205 patients with intertrochanteric fractures.

The lowest point of the fracture line was located in Q1 of the proximal medial wall in 20 patients, with spatial coordinates of (6.44 ± 5.47, 6.14 ± 2.71). In 21 patients, it was in Q2, with coordinates of (−7.23 ± 5.86, 8.31 ± 6.59). In 122 patients, it was in Q3, with coordinates of (−9.59 ± 4.32, −24.43 ± 15.79), and in 42 patients, it was in Q4, with coordinates of (8.18 ± 4.56, −18.20 ± 12.92) (Figure 3).

### 3.2. Diagnostic Performance

The correlation coefficients between the classifications and fracture stability were as follows: Tang classification (*r* = 0.40, 95%CI: 0.30–0.50, *p*  < 0.001), AO classification (*r* = 0.32, 95%CI: 0.22–0.43, *p*  < 0.001), and Evans classification (*r* = 0.38, 95%CI: 0.26–0.49, *p*  < 0.001). The Tang classification showed a stronger correlation with fracture stability than the AO and Evans classifications, effectively reflecting the stability of different fractures.

## 4. Discussion

The present study revealed distinct spatial distribution patterns of fracture lines across four quadrants of the medial wall in intertrochanteric fractures. Comparative analysis demonstrated that the Tang classification system exhibited a superior correlation with medial wall stability compared to both AO and Evans classification systems, effectively reflecting the extent of medial wall damage. This investigation identified that the spatial positioning of the lowest point of the medial wall fracture line within different quadrants demonstrated significant correlation with fracture classification. This spatial parameter critically influences fracture stability. The biomechanical disadvantage arises from the increased moment arm created by the greater distance between the fracture line's lowest point and the femoral head. This spatial relationship generates substantially higher rotational torque during load transmission when the fracture line's lowest point functions as a fulcrum, potentially compromising overall fracture stability. These findings confirm the fundamental hypothesis underlying this research initiative. The results substantiate that precise evaluation of the fracture line's lowest point location provides critical biomechanical insights into medial wall stability, establishing its importance in fracture classification and stability assessment.

Lateral falls, a common cause of hip fractures in the elderly, result in axial stress on the medial side of the femoral head rather than the superior side under normal physiological loading [15, 16]. This biomechanical mismatch in stress distribution frequently results in localized strain overload, ultimately predisposing to cortical failure and fracture initiation. The morphology of the lesser trochanter fragment has been shown to play a pivotal role in characterizing intertrochanteric fractures, particularly in elucidating their biomechanical failure mechanisms [17]. The spatial distribution of the fracture line's lowest point in different quadrants affects the mechanical stability of the hip.

Fractures localized in Q1 and Q2, characterized by preserved lesser trochanter integrity and intact medial wall structures, demonstrated superior mechanical stability. In contrast, Q3 fractures with partial medial wall compromise exhibited intermediate stability, while Q4 fractures with extensive medial wall disruption showed the most compromised biomechanical integrity. The spatial orientation of the fracture line's lowest point significantly influenced stability patterns. When located in Q1 and Q2, the intact medial cortical support effectively maintained structural integrity by preventing proximal fragment buckling and facilitating physiological stress distribution. Specifically, Q1 fractures demonstrated optimal stability due to their characteristic posterior-superior to anterior–inferior fracture orientation. This configuration, combined with intact medial wall support, provided mechanical advantages: the distal fragment resisted anterior rotation of the proximal fragment, while the medial wall prevented varus deformity under axial loading. Consequently, fractures with the lowest point in Q1 exhibit the highest mechanical stability. When the lowest point of the fracture line is located in Q2, the fracture line runs vertically, and the proximal femoral fragment tends to tilt anteriorly. Although the medial wall remains intact, the stability is relatively lower compared to fractures in Q1.

In fractures where the lowest point of the fracture line is located within Q3, concomitant avulsion fractures of the lesser trochanter are frequently observed. Although the medial wall maintains partial structural integrity and retains some capacity to resist varus deformation of the proximal fragment, the increased moment arm resulting from the greater distance between the fracture line's lowest point and the femoral head creates significantly higher rotational torque. This biomechanical disadvantage occurs when the lowest point of the fracture line functions as a fulcrum during load transmission, potentially compromising overall fracture stability despite the preserved medial wall integrity. This leads to a greater tendency for bending, resulting in lower mechanical stability compared to fractures of the lowest point located within Q1 and Q2. When the lowest point of the fracture line is located in Q4, avulsion fractures of the greater trochanter and significant defects in the medial wall are commonly observed, making it difficult to restore the medial wall. Under load applied to the femoral head, the proximal femur loses medial support, leading to ineffective stress transmission and a tendency for the proximal fragment to tilt into varus. As a result, fractures with the lowest point in Q4 exhibit the lowest mechanical stability. Mao et al. [2] and Chang et al. [11] established the anteromedial cortical support concept as a critical framework for intraoperative reduction in trochanteric hip fractures, subsequently developing the Chang reduction quality criteria (CRQC). Importantly, Q4 fracture patterns tend to a poorer CRQC score. In summary, the mechanical stability of intertrochanteric fractures decreases progressively from Q1 to Q4, with Q1 demonstrating the highest stability and Q4 the lowest.

Therefore, an optimal classification system should accurately reflect the mechanical stability of different fracture types to effectively guide clinical decision-making. Due to the overlapping effects in two-dimensional X-ray imaging, which obscure critical radiographic information, the AO and Evans classification systems fail to precisely describe medial wall damage. In contrast, the Tang classification, based on the three-dimensional distribution of fracture lines, effectively captures the extent of medial wall disruption. In our study, by examining the consistency between the risk grading of the AO, Evans, and Tang classifications and the stability grading of the medial wall, we confirmed that the Tang classification most accurately reflects the mechanical stability of the compromised medial wall. Furthermore, Yin et al. [18], through their evaluation of diagnostic consistency across 256 cases of intertrochanteric fractures, concluded that the Tang classification is more reliable than the Evans, AO, and Jensen classifications.

When the lowest point of the fracture is located in Q4, the medial wall is compromised by more than 60%, resulting in significant impairment of medial support and difficulty in reduction. Upon loading of the femoral head, the loss of medial support prevents effective stress transfer from the proximal femur, leading to a tendency for varus collapse of the proximal femur. Q4 fractures predominantly align with Type IV according to the Tang classification system. This fracture type is characterized by diminished medial cortical support and partial involvement of the lateral femoral wall, often resulting in iatrogenic lateral femoral wall fractures. Such iatrogenic complications can transform the fracture from Types IV to V, thereby increasing the complexity of the injury. The medial cortex plays a crucial role in providing medial support, which is essential for preventing femoral head varus. Similarly, an intact lateral wall offers lateral support, effectively preventing lateral displacement of the proximal fragment and medialization of the distal shaft.

Xu et al. [19] proposed the triangular hip stabilization reconstruction theory. They demonstrated that the hip's triangular structure constitutes a stable configuration characterized by a mechanical pattern of two tensile forces, one compressive force, and three mutually constraining forces. Their research revealed that an intact mechanical triangular structure can effectively reduce bending moments, balance the shear forces induced by physiological loads, achieve a balanced stress distribution within the structure, and maintain overall stability. Specifically, the medial wall of the hip mechanics triangle forms an oblique support for the proximal femoral cantilever structure, significantly reducing bending stress and structural deflection. The Tang classification system, which is based on this triangular hip stabilization reconstruction theory, effectively differentiates stability variations among fracture types, thereby providing valuable guidance for clinical decision-making.

Our research had some limitations. This retrospective study may be subject to selection bias, as only patients with preoperative CT were included. Additionally, the homogeneity of our cohort (low-energy fractures) limits generalizability to high-energy trauma patients. Future prospective studies are needed to confirm our findings.

## 5. Conclusion

The extent of medial wall damage in intertrochanteric fractures varies significantly, leading to differences in mechanical stability. The Tang classification is superior to the AO and Evans classifications in differentiating these stability differences, providing effective guidance for clinical practice.

## Data Availability

All data included in this study are available upon request by contact with the corresponding author.
